# Supplementary figures and images for: Genomic insights into the probiotic potential and genes linked to gallic acid metabolism in Pediococcus pentosaceus MBBL6 isolated from healthy cow milk
Source: PLoS One. 2024 Dec 26;19(12):e0316270. doi: 10.1371/journal.pone.0316270 (PMC11671016; doi:10.1371/journal.pone.0316270)

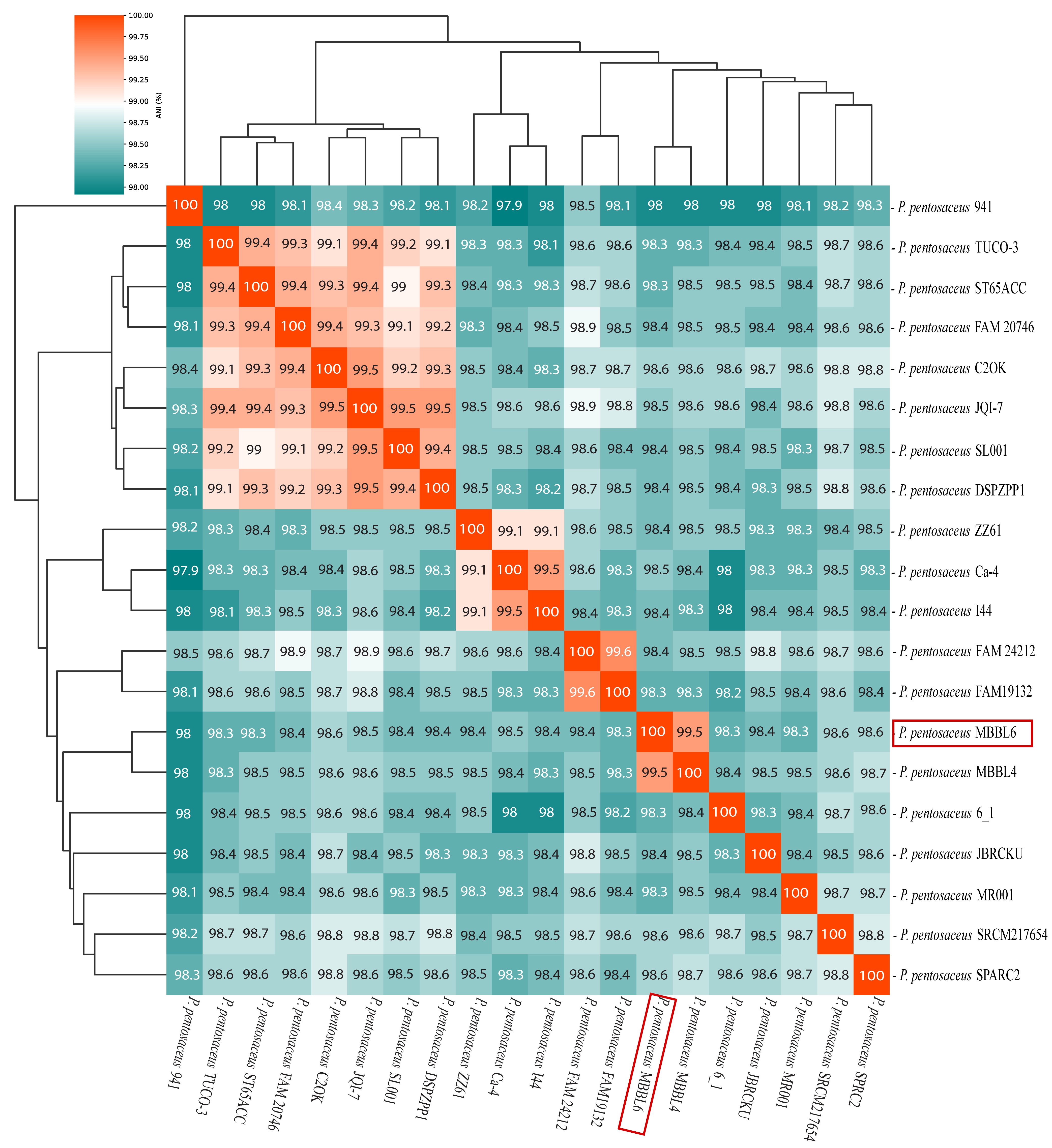

Supplement: S1 Fig — The numbers represent the ANI values (%) between two genome sequences. The proposed species cut-off boundary is above 98%, demonstrating identity with these strains. Study genome is highlighted in red box. (TIFF) [file pone.0316270.s002.tiff]

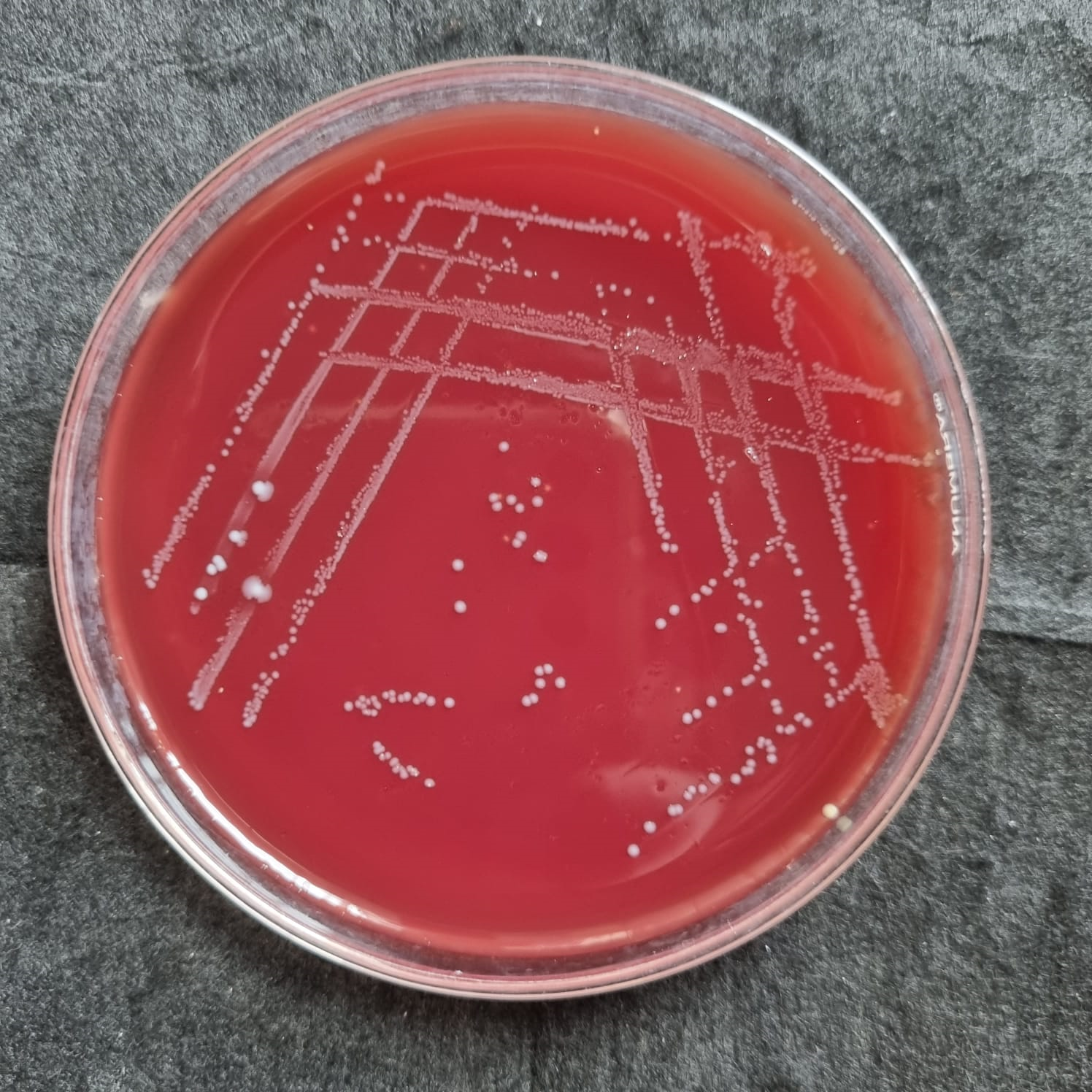

Supplement: S2 Fig — (TIF) [file pone.0316270.s003.tif]

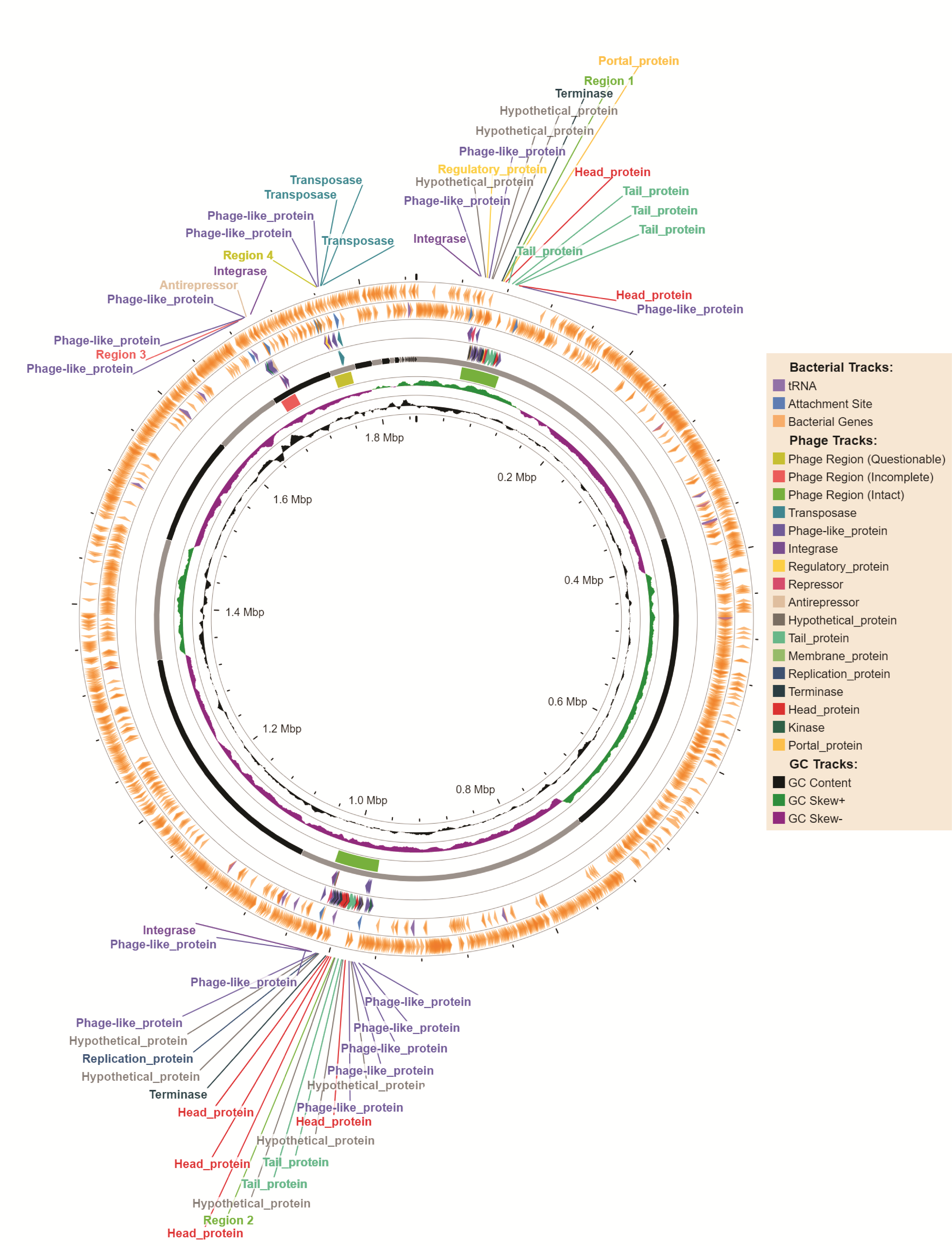

Supplement: S3 Fig — The P. pentosaceus MBBL6 genome harbors two intact (faded green color), one incomplete (faded red color), and one questionable (light yellow color) prophage regions. Circles (from inside to outside) 1 and 2 (GC content and GC skew), circle 3 (phage regions), circles 4 and 5 (phage genes), and circles 6 and 7 (bacterial genes). (TIF) [file pone.0316270.s004.tif]

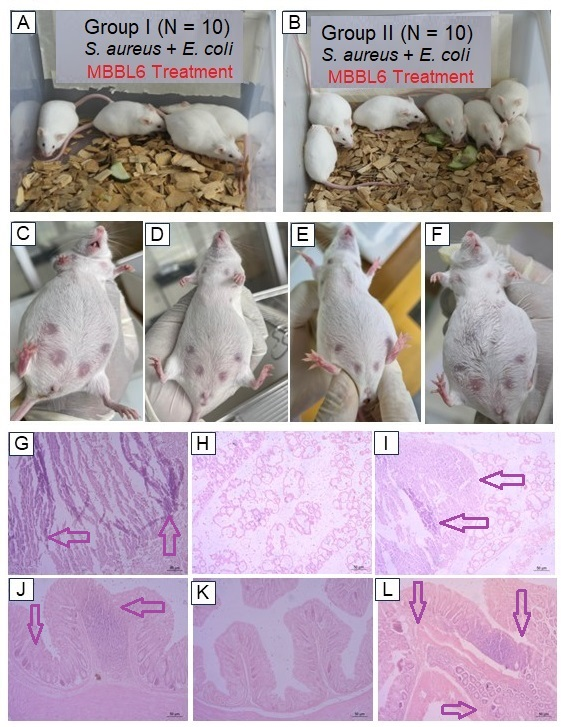

Supplement: S4 Fig — Pathophysiological changes in the mammary glands and gut (colon) of mice after experimental challenge with two major pathogens (S. aureus D4C4 and E. coli G1C5) of bovine mastitis. The mice were challenged at Day 18 of their gestation. (A–B) Two groups of challenged mice. (C) Mouse with induced mastitis showing gross pathological changes in mammary glands (swollen, red and inflamed mammary glands) seven days after challenge. (D) P. pentosaceus MBBL6 treated mouse (at day 7 of treatment) showing mild inflammatory changes in the mammary glands. (E) P. pentosaceus MBBL6 treated mouse (at day 14 of treatment) showing no visible inflammatory changes in the mammary glands. (F) Mouse with induced mastitis receiving only placebo treatment (control). (G–L) Representative photomicrographs of mammary and colon tissues after haematoxylin and eosin (HE) staining. (G and I) The lesions in the mammary alveoli are characterized by a central area of necrosis, broken lobules, damaged acini and destroyed epithelial cells, with large numbers of inflammatory cells, predominantly neutrophils (dark bluish) and macrophages (red) in the mammary lobule, supporting connective tissue and lining of the epithelium. (H) Representative photomicrographs of healthy mammary gland showing no visible inflammatory changes in the epithelium and alveoli after HE staining. (J and L) Representative photomicrographs of colon (crypts, lamina propria, muscularis mucosae and submucosa) tissue after HE staining. Inflammatory changes include moderate to severe inflammatory cell infiltration into mucosa and submucosa, disorder in mucosal structure: Epithelial necrosis, extension of the subepithelial space, and structural damage of villi. (K) Representative photomicrographs of colon tissue of healthy mouse after HE staining where no inflammatory changes were observed. Inflammatory changes in the mammary glands of the mice are mouse are highlighted in purple arrows. Scale bars: 50 μm. (TIF) [file pone.0316270.s005.tif]

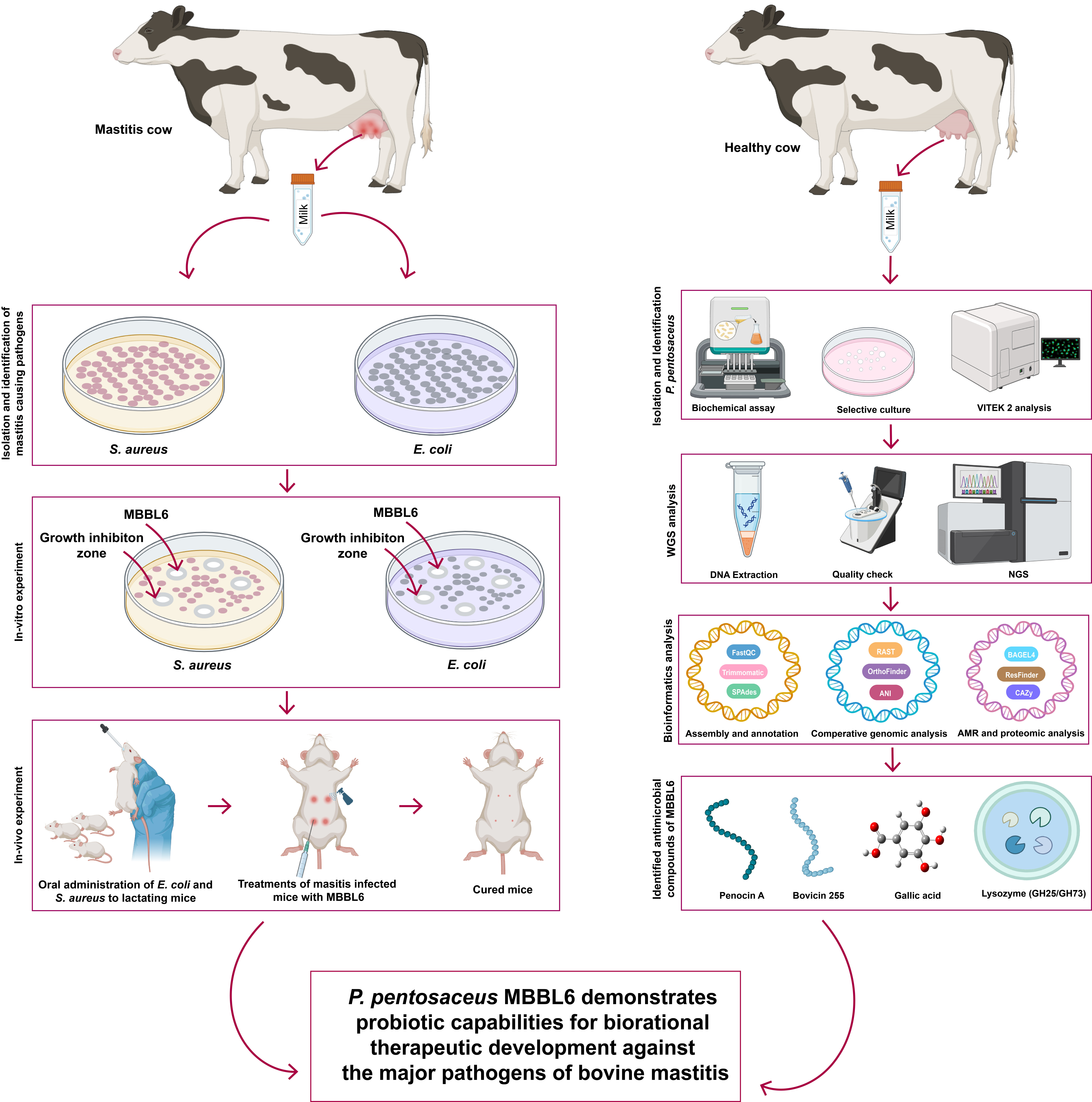

Supplement: S1 Graphical abstract — The study involved sequencing and annotating the genome of P. pentosaceus MBBL6 utilizing advanced bioinformatics tools to elucidate its metabolic pathways, carbohydrate-active enzymes, bacteriocin gene clusters, and secondary metabolite biosynthetic gene clusters. Antimicrobial susceptibility testing of MBBL6 was performed against mastitis-causing pathogens, including S. aureus and E. coli, through both in-vitro and in-vivo trials. Additionally, an in-silico analysis of the core genome was conducted to gain deeper insights into the possible molecular mechanisms by which P. pentosaceus MBBL6 inhibits the growth of these mastitis-causing pathogens. he results indicate P. pentosaceus MBBL6 is a promising probiotic and/or therapeutic agent for mastitis management and biotechnological applications. (TIFF) [file pone.0316270.s016.tiff]
