# Supplementary material for: Genomic insights into the probiotic potential and genes linked to gallic acid metabolism in Pediococcus pentosaceus MBBL6 isolated from healthy cow milk
Source: PLoS One. 2024 Dec 26;19(12):e0316270. doi: 10.1371/journal.pone.0316270 (PMC11671016; doi:10.1371/journal.pone.0316270)
Supplement: S1 Table — (DOCX) [file pone.0316270.s006.docx]

**Table S1.** BioSample ID, isolation source, and country of origin of 19 P. pentosaceus strains.

| **Serial no** | **BioSample ID** | **Strain** | **Isolation source** | **Country** |
| --- | --- | --- | --- | --- |
|  | SAMN33368519 | Ca-4 | Fermented cherry juice | China |
|  | [SAMN27308029](https://www.ncbi.nlm.nih.gov/biosample/SAMN27308029/) | DSPZPP1 | traditional fermented sausages | Italy |
|  | SAMN13612138 | MR001 | shrimp | Thailand |
|  | SAMN11094516 | SL001 | soil | China |
|  | SAMN22933949 | ST65ACC | artisanal raw-milk cheese | Brazil |
|  | SAMN36291298 | ZZ61 | Raw milk | China |
|  | SAMN07701459 | JQI-7 | Fermented dairy | China |
|  | SAMN13008348 | FAM19132 | milk | Switzerland |
|  | SAMN33195126 | SRCM217654 | Cow feces | South Korea |
|  | SAMN16675137 | FAM 20746 | cheese | Switzerland |
|  | SAMN16675142 | FAM 24212 | Milk | Switzerland |
|  | SAMN28159714 | GBRCKU | Healthy buffalo uterus | India |
|  | SAMN10365135 | 941 | milk fermentation | Mali |
|  | SAMN15374531 | TUCO-3 | Cat milk | Chile |
|  | SAMN25610404 | I44 | Human gut | India |
|  | SAMN10721079 | 6_1 | fermented milk | China |
|  | SAMN19473658 | SPARC2 | Animal | Pakistan |
|  | SAMN39454045 | C2OK | Camel gut | Pakistan |
|  | SAMN39456035 | MBBL4 | Cow Milk | Bangladesh |
