# Supplementary material for: Genomic insights into the probiotic potential and genes linked to gallic acid metabolism in Pediococcus pentosaceus MBBL6 isolated from healthy cow milk
Source: PLoS One. 2024 Dec 26;19(12):e0316270. doi: 10.1371/journal.pone.0316270 (PMC11671016; doi:10.1371/journal.pone.0316270)
Supplement: S2 Table — (DOCX) [file pone.0316270.s007.docx]

**Table S2.** Carbohydrate fermentation and enzyme activities of the *P. pentosaceus* MBBL6 isolate.

| Biochemical test | Results |
| --- | --- |
| Maltose | Positive |
| Mannitol | Positive |
| Mannose | Positive |
| Sucrose | Positive |
| Trehalose | Positive |
| N-Acetyl glucosamin | Positive |
| Arginine | Positive |
| Urease | Positive |
| Beta-Glucosidase | Positive |
| Alkaline Phosphatase | Negatative |
| Beta-Glucuronidase | Negatative |
| Beta-Glactosidase | Negatative |
| Indol | Negatative |
| Methyl Red (MR) | Negatative |
| Voges-Proskauer (VP) | Negatative |
| Citrate | Negatative |
| Kligler's Iron Agar (KIA) Test slant | Yellow |
| Kligler's Iron Agar Test Butt | Yellow |
