# Supplementary material for: Genomic insights into the probiotic potential and genes linked to gallic acid metabolism in Pediococcus pentosaceus MBBL6 isolated from healthy cow milk
Source: PLoS One. 2024 Dec 26;19(12):e0316270. doi: 10.1371/journal.pone.0316270 (PMC11671016; doi:10.1371/journal.pone.0316270)
Supplement: S3 Table — (DOCX) [file pone.0316270.s008.docx]

**Table S3.** Prediction of RNAs in P. pentosaceus MBBL6.

| **rRNA** | | | | | |
| --- | --- | --- | --- | --- | --- |
| rRNA ID | Location | Start bp | End bp | Strand | rRNA Type |
| MBBL6.rRNA01 | Chromosome | 370038 | 370149 | - | 5S ribosomal RNA |
| MBBL6.rRNA02 | Chromosome | 333600 | 333711 | - | 5S ribosomal RNA |
| MBBL6.rRNA03 | Chromosome | 281907 | 282018 | - | 5S ribosomal RNA |
| MBBL6.rRNA04 | Chromosome | 3 | 108 | + | 5S ribosomal RNA |
| MBBL6.rRNA05 | Chromosome | 165 | 3084 | + | 23S ribosomal RNA |
| MBBL6.rRNA06 | Chromosome | 3185 | 3296 | + | 5S ribosomal RNA |
| MBBL6.rRNA07 | Chromosome | 82 | 1653 | + | 16S ribosomal RNA |
| **tRNA** | | | | | |
| tRNA ID | Start bp | End bp | Strand | tRNA type | Anti codon |
| MBBL6.tRNA01 | 266044 | 266118 | + | Arg | TCT |
| MBBL6.tRNA02 | 341592 | 341663 | - | Gly | CCC |
| MBBL6.tRNA03 | 351384 | 351468 | - | Leu | CAG |
| MBBL6.tRNA04 | 368237 | 368311 | - | Asp | GTC |
| MBBL6.tRNA05 | 368315 | 368389 | - | fMet | CAT |
| MBBL6.tRNA06 | 368422 | 368494 | - | Glu | TTC |
| MBBL6.tRNA07 | 368517 | 368605 | - | Ser | GCT |
| MBBL6.tRNA08 | 368608 | 368683 | - | Ile | GAT |
| MBBL6.tRNA09 | 368701 | 368772 | - | Gly | TCC |
| MBBL6.tRNA10 | 368796 | 368869 | - | Phe | GAA |
| MBBL6.tRNA11 | 368874 | 368948 | - | Asp | GTC |
| MBBL6.tRNA12 | 368952 | 369026 | - | fMet | CAT |
| MBBL6.tRNA13 | 369035 | 369125 | - | Ser | TGA |
| MBBL6.tRNA14 | 369171 | 369245 | - | Ile2 | CAT |
| MBBL6.tRNA15 | 369265 | 369339 | - | Met | CAT |
| MBBL6.tRNA16 | 369378 | 369452 | - | Pro | TGG |
| MBBL6.tRNA17 | 369461 | 369535 | - | Arg | ACG |
| MBBL6.tRNA18 | 369543 | 369629 | - | Leu | TAA |
| MBBL6.tRNA19 | 369661 | 369733 | - | Gly | GCC |
| MBBL6.tRNA20 | 369743 | 369816 | - | Thr | TGT |
| MBBL6.tRNA21 | 369875 | 369948 | - | Lys | TTT |
| MBBL6.tRNA22 | 369957 | 370030 | - | Val | TAC |
| MBBL6.tRNA23 | 92192 | 92265 | - | Lys | CTT |
| MBBL6.tRNA24 | 115261 | 115334 | + | Glu | CTC |
| MBBL6.tRNA25 | 115342 | 115415 | + | Gln | CTG |
| MBBL6.tRNA26 | 202949 | 203023 | - | Pro | CGG |
| MBBL6.tRNA27 | 203113 | 203187 | - | Arg | ACG |
| MBBL6.tRNA28 | 203208 | 203280 | - | Gly | GCC |
| MBBL6.tRNA29 | 203284 | 203357 | - | Thr | TGT |
| MBBL6.tRNA30 | 203369 | 203451 | - | Leu | TAG |
| MBBL6.tRNA31 | 203493 | 203566 | - | Val | TAC |
| MBBL6.tRNA32 | 279003 | 279077 | - | Asn | GTT |
| MBBL6.tRNA33 | 317864 | 317950 | - | Leu | AAG |
| MBBL6.tRNA34 | 330169 | 330241 | - | Gln | TTG |
| MBBL6.tRNA35 | 330244 | 330330 | - | Tyr | GTA |
| MBBL6.tRNA36 | 333397 | 333470 | - | Thr | CGT |
| MBBL6.tRNA37 | 333518 | 333591 | - | Asn | GTT |
| MBBL6.tRNA38 | 55229 | 55301 | + | Arg | CCG |
| MBBL6.tRNA39 | 281714 | 281788 | - | Thr | GGT |
| MBBL6.tRNA40 | 41070 | 41158 | + | Ser | CGA |
| MBBL6.tRNA41 | 123283 | 123354 | - | Cys | GCA |
| MBBL6.tRNA42 | 123438 | 123522 | - | Leu | CAA |
| MBBL6.tRNA43 | 123569 | 123642 | - | His | GTG |
| MBBL6.tRNA44 | 123647 | 123721 | - | Trp | CCA |
| MBBL6.tRNA45 | 123765 | 123838 | - | Phe | GAA |
| MBBL6.tRNA46 | 123860 | 123934 | - | Asp | GTC |
| MBBL6.tRNA47 | 123952 | 124025 | - | Val | TAC |
| MBBL6.tRNA48 | 124032 | 124104 | - | Glu | TTC |
| MBBL6.tRNA49 | 124122 | 124214 | - | Ser | GGA |
| MBBL6.tRNA50 | 124226 | 124299 | - | Asn | GTT |
| MBBL6.tRNA51 | 22318 | 22392 | + | Arg | CCT |
| MBBL6.tRNA52 | 9070 | 9143 | + | Ala | CGC |
| MBBL6.tRNA53 | 151 | 224 | - | Ala | TGC |
| MBBL6.tRNA54 | 229 | 304 | - | Ile | GAT |
| MBBL6.tRNA55 | 187 | 262 | - | Gln | TTG |
