# Supplementary material for: Genomic insights into the probiotic potential and genes linked to gallic acid metabolism in Pediococcus pentosaceus MBBL6 isolated from healthy cow milk
Source: PLoS One. 2024 Dec 26;19(12):e0316270. doi: 10.1371/journal.pone.0316270 (PMC11671016; doi:10.1371/journal.pone.0316270)
Supplement: S4 Table — (DOCX) [file pone.0316270.s009.docx]

**Table S4.** Carbohydrate metabolism related protein encoding genes predicted in P. pentosaceus MBBL6.

| Gene ID | KO ID | KO Name | KO Description |
| --- | --- | --- | --- |
| gene-V4W90_RS03650 | K00016 | *ldh* | L-lactate dehydrogenase [EC:1.1.1.27] |
| gene-V4W90_RS07420 | K00016 | *ldh* | L-lactate dehydrogenase [EC:1.1.1.27] |
| gene-V4W90_RS05820 | K00134 | *gapA* | glyceraldehyde 3-phosphate dehydrogenase [EC:1.2.1.12] |
| gene-V4W90_RS02590 | K00161 | *pdhA* | pyruvate dehydrogenase E1 component subunit alpha [EC:1.2.4.1] |
| gene-V4W90_RS02595 | K00162 | *pdhB* | pyruvate dehydrogenase E1 component subunit beta [EC:1.2.4.1] |
| gene-V4W90_RS02605 | K00382 | *pdhD* | dihydrolipoyl dehydrogenase [EC:1.8.1.4] |
| gene-V4W90_RS02600 | K00627 | *pdhC* | pyruvate dehydrogenase E2 component [EC:2.3.1.12] |
| gene-V4W90_RS07520 | K00850 | *pfkA* | 6-phosphofructokinase 1 [EC:2.7.1.11] |
| gene-V4W90_RS07520 | K00873 | *pyk* | pyruvate kinase [EC:2.7.1.40] |
| gene-V4W90_RS05815 | K00927 | *pgk* | phosphoglycerate kinase [EC:2.7.2.3] |
| gene-V4W90_RS08540 | K01223 | *bglA* | 6-phospho-beta-glucosidase [EC:3.2.1.86] |
| gene-V4W90_RS02575 | K01223 | *bglA* | 6-phospho-beta-glucosidase [EC:3.2.1.86] |
| gene-V4W90_RS06690 | K01223 | *bglA* | 6-phospho-beta-glucosidase [EC:3.2.1.86] |
| gene-V4W90_RS07245 | K01223 | *bglA* | 6-phospho-beta-glucosidase [EC:3.2.1.86] |
| gene-V4W90_RS04340 | K01624 | *fbaA* | fructose-bisphosphate aldolase, class II [EC:4.1.2.13] |
| gene-V4W90_RS05805 | K01689 | *eno* | enolase 1/2/3 [EC:4.2.1.11] |
| gene-V4W90_RS00165 | K01785 | *galM* | aldose 1-epimerase [EC:5.1.3.3] |
| gene-V4W90_RS07345 | K01785 | *galM* | aldose 1-epimerase [EC:5.1.3.3] |
| gene-V4W90_RS07435 | K01785 | *galM* | aldose 1-epimerase [EC:5.1.3.3] |
| gene-V4W90_RS05810 | K01803 | *tpiA* | triosephosphate isomerase (TIM) [EC:5.3.1.1] |
| gene-V4W90_RS04330 | K01810 | *pgi* | glucose-6-phosphate isomerase [EC:5.3.1.9] |
| gene-V4W90_RS05430 | K01834 | *gpmA* | 2,3-bisphosphoglycerate-dependent phosphoglycerate mutase [EC:5.4.2.11] |
| gene-V4W90_RS02910 | K01834 | *gpmA* | 2,3-bisphosphoglycerate-dependent phosphoglycerate mutase [EC:5.4.2.11] |
| gene-V4W90_RS05885 | K01835 | *pgm* | phosphoglucomutase [EC:5.4.2.2] |
| gene-V4W90_RS08795 | K02777 | *crr* | sugar PTS system EIIA component [EC:2.7.1.-] |
| gene-V4W90_RS07075 | K04041 | *fbp4* | fructose-1,6-bisphosphatase III [EC:3.1.3.11] |
| gene-V4W90_RS01485 | K25026 | *glk* | glucokinase [EC:2.7.1.2] |
| gene-V4W90_RS07380 | K01679 | *fumC* | fumarate hydratase, class II [EC:4.2.1.2] |
| gene-V4W90_RS06875 | K01958 | *pyc* | pyruvate carboxylase [EC:6.4.1.1] |
| gene-V4W90_RS01580 | K00033 | *gnd* | 6-phosphogluconate dehydrogenase [EC:1.1.1.44 1.1.1.343] |
| gene-V4W90_RS04390 | K00033 | *gnd* | 6-phosphogluconate dehydrogenase [EC:1.1.1.44 1.1.1.343] |
| gene-V4W90_RS08815 | K00034 | *gdh* | glucose 1-dehydrogenase [EC:1.1.1.47] |
| gene-V4W90_RS03925 | K00036 | *zwf* | glucose-6-phosphate 1-dehydrogenase [EC:1.1.1.49 1.1.1.363] |
| gene-V4W90_RS07220 | K00615 | *tktA* | transketolase [EC:2.2.1.1] |
| gene-V4W90_RS07225 | K00616 | *talB* | transaldolase [EC:2.2.1.2] |
| gene-V4W90_RS03785 | K00852 | *rbsK* | ribokinase [EC:2.7.1.15] |
| gene-V4W90_RS02965 | K00852 | *rbsK* | ribokinase [EC:2.7.1.15] |
| gene-V4W90_RS06630 | K00948 | *prsA* | ribose-phosphate pyrophosphokinase [EC:2.7.6.1] |
| gene-V4W90_RS07890 | K00948 | *prsA* | ribose-phosphate pyrophosphokinase [EC:2.7.6.1] |
| gene-V4W90_RS06340 | K01621 | *xfp* | xylulose-5-phosphate/fructose-6-phosphate phosphoketolase [EC:4.1.2.9 4.1.2.22] |
| gene-V4W90_RS01290 | K01783 | *rpe* | ribulose-phosphate 3-epimerase [EC:5.1.3.1] |
| gene-V4W90_RS05410 | K01807 | *rpiA* | ribose 5-phosphate isomerase A [EC:5.3.1.6] |
| gene-V4W90_RS08040 | K07404 | *pgl* | 6-phosphogluconolactonase [EC:3.1.1.31] |
| gene-V4W90_RS04385 | K25031 | *gntK* | gluconokinase [EC:2.7.1.12] |
| gene-V4W90_RS07155 | K00853 | *araB* | L-ribulokinase [EC:2.7.1.16] |
| gene-V4W90_RS07270 | K00854 | *xylB* | xylulokinase [EC:2.7.1.17] |
| gene-V4W90_RS05895 | K00963 | *galU* | UTP--glucose-1-phosphate uridylyltransferase [EC:2.7.7.9] |
| gene-V4W90_RS07165 | K01804 | *araA* | L-arabinose isomerase [EC:5.3.1.4] |
| gene-V4W90_RS07265 | K01805 | *xylA* | xylose isomerase [EC:5.3.1.5] |
| gene-V4W90_RS07160 | K03077 | *araD* | L-ribulose-5-phosphate 4-epimerase [EC:5.1.3.4] |
| gene-V4W90_RS04815 | K00847 | *scrK* | fructokinase [EC:2.7.1.4] |
| gene-V4W90_RS08525 | K00847 | *scrK* | fructokinase [EC:2.7.1.4] |
| gene-V4W90_RS07875 | K00882 | *fruK* | 1-phosphofructokinase [EC:2.7.1.56] |
| gene-V4W90_RS04255 | K01809 | *manA* | mannose-6-phosphate isomerase [EC:5.3.1.8] |
| gene-V4W90_RS05445 | K02769 | *fruAb* | fructose PTS system EIIB component [EC:2.7.1.202] |
| gene-V4W90_RS07870 | K02770 | *fruA* | fructose PTS system EIIBC or EIIC component [EC:2.7.1.202] |
| gene-V4W90_RS08035 | K02781 | *srlB* | glucitol/sorbitol PTS system EIIA component [EC:2.7.1.198] |
| gene-V4W90_RS02645 | K02793 | *manXa* | mannose PTS system EIIA component [EC:2.7.1.191] |
| gene-V4W90_RS05470 | K02794 | *manX* | mannose PTS system EIIAB component [EC:2.7.1.191] |
| gene-V4W90_RS02665 | K02795 | *manY* | mannose PTS system EIIC component |
| gene-V4W90_RS05465 | K02795 | *manY* | mannose PTS system EIIC component |
| gene-V4W90_RS05460 | K02796 | *manZ* | mannose PTS system EIID component |
| gene-V4W90_RS02670 | K02796 | *manZ* | mannose PTS system EIID component |
| gene-V4W90_RS06935 | K00849 | *galK* | galactokinase [EC:2.7.1.6] |
| gene-V4W90_RS07365 | K00849 | *galK* | galactokinase [EC:2.7.1.6] |
| gene-V4W90_RS07375 | K00965 | *galT* | UDPglucose--hexose-1-phosphate uridylyltransferase [EC:2.7.7.12] |
| gene-V4W90_RS08500 | K01187 | *malZ* | alpha-glucosidase [EC:3.2.1.20] |
| gene-V4W90_RS07360 | K01190 | *lacZ* | beta-galactosidase [EC:3.2.1.23] |
| gene-V4W90_RS07355 | K01190 | *lacZ* | beta-galactosidase [EC:3.2.1.23] |
| gene-V4W90_RS08510 | K01193 | *sacA* | beta-fructofuranosidase [EC:3.2.1.26] |
| gene-V4W90_RS07070 | K01635 | *lacD* | tagatose 1,6-diphosphate aldolase [EC:4.1.2.40] |
| gene-V4W90_RS04975 | K01784 | *galE* | UDP-glucose 4-epimerase [EC:5.1.3.2] |
| gene-V4W90_RS07370 | K01784 | *galE* | UDP-glucose 4-epimerase [EC:5.1.3.2] |
| gene-V4W90_RS08325 | K01854 | *glf* | UDP-galactopyranose mutase [EC:5.4.99.9] |
| gene-V4W90_RS02680 | K02082 | *agaS* | D-galactosamine 6-phosphate deaminase/isomerase [EC:3.5.99.-] |
| gene-V4W90_RS08520 | K07407 | *galA* | alpha-galactosidase [EC:3.2.1.22] |
| gene-V4W90_RS05580 | K07407 | *galA* | alpha-galactosidase [EC:3.2.1.22] |
| gene-V4W90_RS07205 | K02821 | *ulaC* | ascorbate PTS system EIIA or EIIAB component [EC:2.7.1.194] |
| gene-V4W90_RS07210 | K02822 | *sgaB* | ascorbate PTS system EIIB component [EC:2.7.1.194] |
| gene-V4W90_RS07215 | K03475 | *ulaA* | ascorbate PTS system EIIC component |
| gene-V4W90_RS00620 | K00691 | *mapA* | maltose phosphorylase [EC:2.4.1.8] |
| gene-V4W90_RS02430 | K01226 | *treC* | trehalose-6-phosphate hydrolase [EC:3.2.1.93] |
| gene-V4W90_RS00625 | K01838 | *pgmB* | beta-phosphoglucomutase [EC:5.4.2.6] |
| gene-V4W90_RS06695 | K02759 | *celC* | cellobiose PTS system EIIA component [EC:2.7.1.196 2.7.1.205] |
| gene-V4W90_RS06700 | K02760 | *chbB* | cellobiose PTS system EIIB component [EC:2.7.1.196 2.7.1.205] |
| gene-V4W90_RS08725 | K02761 | *chbC* | cellobiose PTS system EIIC component |
| gene-V4W90_RS07490 | K02761 | *chbC* | cellobiose PTS system EIIC component |
| gene-V4W90_RS02580 | K02761 | *chbC* | cellobiose PTS system EIIC component |
| gene-V4W90_RS00235 | K02810 | *scrA* | sucrose PTS system EIIBCA or EIIBC component [EC:2.7.1.211] |
| gene-V4W90_RS08515 | K02810 | *scrA* | sucrose PTS system EIIBCA or EIIBC component [EC:2.7.1.211] |
| gene-V4W90_RS05745 | K00075 | *murB* | UDP-N-acetylmuramate dehydrogenase [EC:1.3.1.98] |
| gene-V4W90_RS03570 | K00790 | *murA* | UDP-N-acetylglucosamine 1-carboxyvinyltransferase [EC:2.5.1.7] |
| gene-V4W90_RS05725 | K00820 | *glmS* | glutamine---fructose-6-phosphate transaminase (isomerizing) [EC:2.6.1.16] |
| gene-V4W90_RS05520 | K01443 | *nagA* | N-acetylglucosamine-6-phosphate deacetylase [EC:3.5.1.25] |
| gene-V4W90_RS02685 | K01443 | *nagA* | N-acetylglucosamine-6-phosphate deacetylase [EC:3.5.1.25] |
| gene-V4W90_RS07115 | K01788 | *nanE* | N-acylglucosamine-6-phosphate 2-epimerase [EC:5.1.3.9] |
| gene-V4W90_RS08235 | K01791 | *wecB* | UDP-N-acetylglucosamine 2-epimerase (non-hydrolysing) [EC:5.1.3.14] |
| gene-V4W90_RS06430 | K02564 | *nagB* | glucosamine-6-phosphate deaminase [EC:3.5.99.6] |
| gene-V4W90_RS02385 | K02804 | *nagE* | N-acetylglucosamine PTS system EIICBA or EIICB component [EC:2.7.1.193] |
| gene-V4W90_RS05730 | K03431 | *glmM* | phosphoglucosamine mutase [EC:5.4.2.10] |
| gene-V4W90_RS06640 | K04042 | *glmU* | bifunctional UDP-N-acetylglucosamine pyrophosphorylase / glucosamine-1-phosphate N-acetyltransferase [EC:2.7.7.23 2.3.1.157] |
| gene-V4W90_RS00230 | K07106 | *murQ* | N-acetylmuramic acid 6-phosphate etherase [EC:4.2.1.126] |
| gene-V4W90_RS01945 | K00158 | *poxL* | pyruvate oxidase [EC:1.2.3.3] |
| gene-V4W90_RS03850 | K00158 | *poxL* | pyruvate oxidase [EC:1.2.3.3] |
| gene-V4W90_RS06420 | K00625 | *pta* | phosphate acetyltransferase [EC:2.3.1.8] |
| gene-V4W90_RS06425 | K00925 | *ackA* | acetate kinase [EC:2.7.2.1] |
| gene-V4W90_RS02355 | K00925 | *ackA* | acetate kinase [EC:2.7.2.1] |
| gene-V4W90_RS01560 | K01512 | *acyP* | acylphosphatase [EC:3.6.1.7] |
| gene-V4W90_RS01155 | K01961 | *accC* | acetyl-CoA carboxylase, biotin carboxylase subunit [EC:6.4.1.2 6.3.4.14] |
| gene-V4W90_RS01145 | K01962 | *accA* | acetyl-CoA carboxylase carboxyl transferase subunit alpha [EC:6.4.1.2 2.1.3.15] |
| gene-V4W90_RS01150 | K01963 | *accD* | acetyl-CoA carboxylase carboxyl transferase subunit beta [EC:6.4.1.2 2.1.3.15] |
| gene-V4W90_RS01165 | K02160 | *accB* | acetyl-CoA carboxylase biotin carboxyl carrier protein |
| gene-V4W90_RS01090 | K03778 | *ldhA* | D-lactate dehydrogenase [EC:1.1.1.28] |
| gene-V4W90_RS03365 | K22212 | *mleA* | malolactic enzyme [EC:4.1.1.101] |
| gene-V4W90_RS02990 | K22373 | *larA* | lactate racemase [EC:5.1.2.1] |
| gene-V4W90_RS02110 | K23257 | *yvgN* | methylglyoxal/glyoxal reductase [EC:1.1.1.283 1.1.1.-] |
| gene-V4W90_RS04220 | K00600 | *glyA* | glycine hydroxymethyltransferase [EC:2.1.2.1] |
| gene-V4W90_RS00680 | K00865 | *glxK* | glycerate 2-kinase [EC:2.7.1.165] |
| gene-V4W90_RS01450 | K01915 | *glnA* | glutamine synthetase [EC:6.3.1.2] |
| gene-V4W90_RS06725 | K00086 | *dhaT* | 1,3-propanediol dehydrogenase [EC:1.1.1.202] |
| gene-V4W90_RS05520 | K01443 | *nagA* | N-acetylglucosamine-6-phosphate deacetylase [EC:3.5.1.25] |
| gene-V4W90_RS02685 | K01443 | *nagA* | N-acetylglucosamine-6-phosphate deacetylase [EC:3.5.1.25] |
| gene-V4W90_RS07115 | K01788 | *nanE* | N-acylglucosamine-6-phosphate 2-epimerase [EC:5.1.3.9] |
| gene-V4W90_RS08235 | K01791 | *wecB* | UDP-N-acetylglucosamine 2-epimerase (non-hydrolysing) [EC:5.1.3.14] |
| gene-V4W90_RS06430 | K02564 | *nagB* | glucosamine-6-phosphate deaminase [EC:3.5.99.6] |
| gene-V4W90_RS02385 | K02804 | *nagE* | N-acetylglucosamine PTS system EIICBA or EIICB component [EC:2.7.1.193] |
| gene-V4W90_RS05730 | K03431 | *glmM* | phosphoglucosamine mutase [EC:5.4.2.10] |
| gene-V4W90_RS06640 | K04042 | *glmU* | bifunctional UDP-N-acetylglucosamine pyrophosphorylase / glucosamine-1-phosphate N-acetyltransferase [EC:2.7.7.23 2.3.1.157] |
