# Supplementary material for: Genomic insights into the probiotic potential and genes linked to gallic acid metabolism in Pediococcus pentosaceus MBBL6 isolated from healthy cow milk
Source: PLoS One. 2024 Dec 26;19(12):e0316270. doi: 10.1371/journal.pone.0316270 (PMC11671016; doi:10.1371/journal.pone.0316270)
Supplement: S5 Table — (DOCX) [file pone.0316270.s010.docx]

**Table S5.** Probiotic and vitamin B biosynthesis related genes predicted in *P. pentosaceus* MBBL6.

| **Probiotic related genes** | | | | |
| --- | --- | --- | --- | --- |
| Predicted Function | Gene ID | KO ID | KO Name | KO Description |
| Acid tolerance | gene-V4W90_RS06695 | K02759 | *celC, chbA* | cellobiose PTS system EIIA component [EC:2.7.1.196 2.7.1.205] |
|  | gene-V4W90_RS06700 | K02760 | *celA, chbB* | cellobiose PTS system EIIB component [EC:2.7.1.196 2.7.1.205] |
|  | gene-V4W90_RS08725 | K02761 | *celB, chbC* | cellobiose PTS system EIIC component |
|  | gene-V4W90_RS07490 | K02761 | *celB, chbC* | cellobiose PTS system EIIC component |
|  | gene-V4W90_RS02580 | K02761 | *celB, chbC* | cellobiose PTS system EIIC component |
|  | gene-V4W90_RS04200 | K02108 | *ATPF0A, atpB* | F-type H+-transporting ATPase subunit a |
|  | gene-V4W90_RS04195 | K02109 | *ATPF0B, atpF* | F-type H+-transporting ATPase subunit b |
|  | gene-V4W90_RS04195 | K02110 | *ATPF0C, atpE* | F-type H+-transporting ATPase subunit c |
|  | gene-V4W90_RS04180 | K02111 | *ATPF1A, atpA* | F-type H+/Na+-transporting ATPase subunit alpha [EC:7.1.2.2 7.2.2.1] |
|  | gene-V4W90_RS04170 | K02112 | *ATPF1B, atpD* | F-type H+/Na+-transporting ATPase subunit beta [EC:7.1.2.2 7.2.2.1] |
|  | gene-V4W90_RS04185 | K02113 | *ATPF1D, atpH* | F-type H+-transporting ATPase subunit delta |
|  | gene-V4W90_RS04165 | K02114 | *ATPF1E, atpC* | F-type H+-transporting ATPase subunit epsilon |
|  | gene-V4W90_RS04175 | K02115 | *ATPF1G, atpG* | F-type H+-transporting ATPase subunit gamma |
| Antioxidant | gene-V4W90_RS05890 | K00384 | *trxB, TRR* | thioredoxin reductase (NADPH) [EC:1.8.1.9] |
|  | gene-V4W90_RS04070 | K08968 | *msrC* | L-methionine (R)-S-oxide reductase [EC:1.8.4.14] |
|  | gene-V4W90_RS01580 | K00033 | *PGD, gnd, gntZ* | 6-phosphogluconate dehydrogenase [EC:1.1.1.44 1.1.1.343] |
|  | gene-V4W90_RS04390 | K00033 | *PGD, gnd, gntZ* | 6-phosphogluconate dehydrogenase [EC:1.1.1.44 1.1.1.343] |
|  | gene-V4W90_RS03925 | K00036 | *G6PD, zwf* | glucose-6-phosphate 1-dehydrogenase [EC:1.1.1.49 1.1.1.363] |
|  | gene-V4W90_RS06885 | K00383 | *GSR, gor* | glutathione reductase (NADPH) [EC:1.8.1.7] |
|  | gene-V4W90_RS07120 | K00383 | *GSR, gor* | glutathione reductase (NADPH) [EC:1.8.1.7] |
|  | gene-V4W90_RS06245 | k03671 | *TXN, trxA* | thioredoxin |
|  | gene-V4W90_RS03880 | k03671 | *TXN, trxA* | thioredoxin |
|  | gene-V4W90_RS07015 | K00215 | *dapB* | 4-hydroxy-tetrahydrodipicolinate reductase [EC:1.17.1.8] |
| Immunity | gene-V4W90_RS01935 | K00789 | *metK, MAT* | S-adenosylmethionine synthetase [EC:2.5.1.6] |
| heat shock/stress | gene-V4W90_RS07535 | K03695 | *clpB* | ATP-dependent Clp protease ATP-binding subunit ClpB |
|  | gene-V4W90_RS07755 | K03544 | *clpX, CLPX* | ATP-dependent Clp protease ATP-binding subunit ClpX |
|  | gene-V4W90_RS05840 | K01358 | *clpP, CLPP* | ATP-dependent Clp protease, protease subunit [EC:3.4.21.92] |
|  | gene-V4W90_RS03970 | K03553 | *recA* | recombination protein RecA |
|  | gene-V4W90_RS03235 | K13993 | *HSP20* | HSP20 family protein |
|  | gene-V4W90_RS06005 | K04077 | *groEL, HSPD1* | chaperonin GroEL [EC:5.6.1.7] |
|  | gene-V4W90_RS00990 | K04043 | *dnaK, HSPA9* | molecular chaperone DnaK |
|  | gene-V4W90_RS05840 | K01358 | *clpP, CLPP* | ATP-dependent Clp protease, protease subunit [EC:3.4.21.92] |
| surface adhesion | gene-V4W90_RS06275 | K19005 | *ltaS* | lipoteichoic acid synthase [EC:2.7.8.20] |
|  | gene-V4W90_RS08200 | K19005 | *ltaS* | lipoteichoic acid synthase [EC:2.7.8.20] |
|  | gene-V4W90_RS05805 | K01689 | *ENO1_2_3, eno* | enolase 1/2/3 [EC:4.2.1.11] |
|  | gene-V4W90_RS05820 | K00134 | *GAPDH, gapA* | glyceraldehyde 3-phosphate dehydrogenase (phosphorylating) [EC:1.2.1.12] |
|  | gene-V4W90_RS01565 | K03217 | *yidC, spoIIIJ, OXA1, ccfA* | YidC/Oxa1 family membrane protein insertase |
|  | gene-V4W90_RS07765 | K02358 | *tuf, TUFM* | elongation factor Tu |
|  | gene-V4W90_RS02245 | K03217 | *yidC, spoIIIJ, OXA1, ccfA* | YidC/Oxa1 family membrane protein insertase |
| **Vitamin B biosynthesis related genes** | | | | |
| Vitamin name | Gene ID | KO ID | KO Name | KO Description |
| Thiamine | gene-V4W90_RS08425 | K00788 | *thiE* | thiamine-phosphate pyrophosphorylase [EC:2.5.1.3] |
|  | gene-V4W90_RS08435 | K00878 | *thiM* | hydroxyethylthiazole kinase [EC:2.7.1.50] |
|  | gene-V4W90_RS04595 | K00939 | *adk, AK* | adenylate kinase [EC:2.7.4.3] |
|  | gene-V4W90_RS07475 | K00941 | *thiD* | hydroxymethylpyrimidine/phosphomethylpyrimidine kinase [EC:2.7.1.49 2.7.4.7] |
|  | gene-V4W90_RS08430 | K00941 | *thiD* | hydroxymethylpyrimidine/phosphomethylpyrimidine kinase [EC:2.7.1.49 2.7.4.7] |
|  | gene-V4W90_RS01285 | K00949 | *thiN, TPK1, THI80* | thiamine pyrophosphokinase [EC:2.7.6.2] |
|  | gene-V4W90_RS02775 | K01077 | *E3.1.3.1, phoA, phoB* | alkaline phosphatase [EC:3.1.3.1] |
|  | gene-V4W90_RS04055 | K03151 | *thiI* | tRNA uracil 4-sulfurtransferase [EC:2.8.1.4] |
|  | gene-V4W90_RS08420 | K03707 | *tenA* | thiaminase (transcriptional activator TenA) [EC:3.5.99.2] |
|  | gene-V4W90_RS07920 | K04487 | *iscS, NFS1* | cysteine desulfurase [EC:2.8.1.7] |
|  | gene-V4W90_RS04060 | K04487 | *iscS, NFS1* | cysteine desulfurase [EC:2.8.1.7] |
|  | gene-V4W90_RS01295 | K06949 | *rsgA, engC* | ribosome biogenesis GTPase / thiamine phosphate phosphatase [EC:3.6.1.- 3.1.3.100] |
| Pyridoxin | gene-V4W90_RS01805 | K05275 | *E1.1.1.65* | pyridoxine 4-dehydrogenase [EC:1.1.1.65] |
| Nicotinamide | gene-V4W90_RS02360 | K00135 | *gabD* | succinate-semialdehyde dehydrogenase / glutarate-semialdehyde dehydrogenase [EC:1.2.1.16 1.2.1.79 1.2.1.20] |
|  | gene-V4W90_RS05505 | K00763 | *pncB, NAPRT1* | nicotinate phosphoribosyltransferase [EC:6.3.4.21] |
|  | gene-V4W90_RS08060 | K00858 | *ppnK, NADK* | NAD+ kinase [EC:2.7.1.23] |
|  | gene-V4W90_RS01615 | K00969 | *nadD* | nicotinate-nucleotide adenylyltransferase [EC:2.7.7.18] |
|  | gene-V4W90_RS05500 | K01916 | *nadE* | NAD+ synthase [EC:6.3.1.5] |
| Pantothenate | gene-V4W90_RS01695 | K00859 | *coaE* | dephospho-CoA kinase [EC:2.7.1.24] |
|  | gene-V4W90_RS06075 | K00867 | *coaA* | type I pantothenate kinase [EC:2.7.1.33] |
|  | gene-V4W90_RS07810 | K00954 | *E2.7.7.3A, coaD, kdtB* | pantetheine-phosphate adenylyltransferase [EC:2.7.7.3] |
|  | gene-V4W90_RS03620 | K00997 | *acpS* | holo-[acyl-carrier protein] synthase [EC:2.7.8.7] |
|  | gene-V4W90_RS06085 | K01652 | *E2.2.1.6L, ilvB, ilvG, ilvI* | acetolactate synthase I/II/III large subunit [EC:2.2.1.6] |
|  | gene-V4W90_RS00700 | K01652 | *E2.2.1.6L, ilvB, ilvG, ilvI* | acetolactate synthase I/II/III large subunit [EC:2.2.1.6] |
|  | gene-V4W90_RS01325 | K13038 | *coaBC, dfp* | phosphopantothenoylcysteine decarboxylase / phosphopantothenate---cysteine ligase [EC:4.1.1.36 6.3.2.5] |
| Biotin | gene-V4W90_RS00960 | K00059 | *fabG, OAR1* | 3-oxoacyl-[acyl-carrier protein] reductase [EC:1.1.1.100] |
|  | gene-V4W90_RS03990 | K00059 | *fabG, OAR1* | 3-oxoacyl-[acyl-carrier protein] reductase [EC:1.1.1.100] |
|  | gene-V4W90_RS02105 | K00059 | *fabG, OAR1* | 3-oxoacyl-[acyl-carrier protein] reductase [EC:1.1.1.100] |
|  | gene-V4W90_RS01175 | K00059 | *fabG, OAR1* | 3-oxoacyl-[acyl-carrier protein] reductase [EC:1.1.1.100] |
|  | gene-V4W90_RS01140 | K00208 | *fabI* | enoyl-[acyl-carrier protein] reductase I [EC:1.3.1.9 1.3.1.10] |
|  | gene-V4W90_RS01160 | K02372 | *fabZ* | 3-hydroxyacyl-[acyl-carrier-protein] dehydratase [EC:4.2.1.59] |
|  | gene-V4W90_RS01195 | K02372 | *fabZ* | 3-hydroxyacyl-[acyl-carrier-protein] dehydratase [EC:4.2.1.59] |
|  | gene-V4W90_RS03270 | K03524 | *birA* | biotin operon repressor / biotin---[acetyl-CoA-carboxylase] ligase [EC:6.3.4.15] |
|  | gene-V4W90_RS01170 | K09458 | *fabF, OXSM, CEM1* | 3-oxoacyl-[acyl-carrier-protein] synthase II [EC:2.3.1.179] |
| Folate | gene-V4W90_RS00115 | K00287 | *DHFR, folA* | dihydrofolate reductase [EC:1.5.1.3] |
|  | gene-V4W90_RS02775 | K01077 | *E3.1.3.1, phoA, phoB* | alkaline phosphatase [EC:3.1.3.1] |
|  | gene-V4W90_RS04040 | K11754 | *folC* | dihydrofolate synthase / folylpolyglutamate synthase [EC:6.3.2.12 6.3.2.17] |
|  | gene-V4W90_RS02375 | K14652 | *ribBA* | 3,4-dihydroxy 2-butanone 4-phosphate synthase / GTP cyclohydrolase II [EC:4.1.99.12 3.5.4.25] |
