# Supplementary material for: Genomic insights into the probiotic potential and genes linked to gallic acid metabolism in Pediococcus pentosaceus MBBL6 isolated from healthy cow milk
Source: PLoS One. 2024 Dec 26;19(12):e0316270. doi: 10.1371/journal.pone.0316270 (PMC11671016; doi:10.1371/journal.pone.0316270)
Supplement: S6 Table — (DOCX) [file pone.0316270.s011.docx]

**Table S6.** Prediction of primary metabolic and secondary metabolite biosynthesis gene clusters in *P. pentosaceus* MBBL6.

| Region | Type | From | To | Most similar known clusters |
| --- | --- | --- | --- | --- |
| Primary metabolic region 1 | Arginine2_Hcarbonate | 244,703 | 267,923 | Arginine to hydrogen carbonate *P. aeruginosa*, ARG (100% similarity) |
| Primary metabolic region 2 | gallic_acid_met | 255,055 | 277,148 | Gallic acid degradation *B. sp.* KLE, GALL (100% similarity) |
| Secondary metabolic region 1 | T3PKS | 193,545 | 234,711 | fusaricidin B, (25% of genes show similarity), xantholipin (4% of genes show similarity) |
| Secondary metabolic region 1 | RiPP-like | 36,717 | 46,899 | - |
