# Supplementary material for: Genomic insights into the probiotic potential and genes linked to gallic acid metabolism in Pediococcus pentosaceus MBBL6 isolated from healthy cow milk
Source: PLoS One. 2024 Dec 26;19(12):e0316270. doi: 10.1371/journal.pone.0316270 (PMC11671016; doi:10.1371/journal.pone.0316270)
Supplement: S7 Table — The third column in the right column represents zone of inhibition (ZoI). (DOCX) [file pone.0316270.s012.docx]

**Table S7.** Antibiotic susceptibility results from culture plate: R = Resistance, S = Susceptible, I = intermediate AST results. The third column in the right column represents zone of inhibition (ZoI).

| Antibiotic | AST result | ZoI |
| --- | --- | --- |
| Azithromycin (AZM) | R | 0 |
| Amikacin (AK) | S | 23 |
| Cefepime (FEP) | S | 28 |
| Ceftriaxone (CRO) | S | 28 |
| Cefuroxim sodium (CXM) | S | 25 |
| Ciprofloxacin (CIP) | S | 32 |
| Gentamicin (CN) | S | 25 |
| Nalidixic acid (NA) | R | 0 |
| Imipenem (IPM) | S | 44 |
| Meropenem (MEM) | S | 26 |
