# Supplementary material for: Genomic insights into the probiotic potential and genes linked to gallic acid metabolism in Pediococcus pentosaceus MBBL6 isolated from healthy cow milk
Source: PLoS One. 2024 Dec 26;19(12):e0316270. doi: 10.1371/journal.pone.0316270 (PMC11671016; doi:10.1371/journal.pone.0316270)
Supplement: S8 Table — (DOCX) [file pone.0316270.s013.docx]

**Table S8.** Prediction of antibiotic resistance genes in P. pentosaceus MBBL6.

| ARGs | Start | End | Strand | Drug resistance |
| --- | --- | --- | --- | --- |
| *lnuA* | 15985 | 16470 | + | Lincosamide |
| *ErmB* | 706 | 1451 | - | Lincosamide, Macrolide and Streptogramin |
