# Supplementary material for: Genomic insights into the probiotic potential and genes linked to gallic acid metabolism in Pediococcus pentosaceus MBBL6 isolated from healthy cow milk
Source: PLoS One. 2024 Dec 26;19(12):e0316270. doi: 10.1371/journal.pone.0316270 (PMC11671016; doi:10.1371/journal.pone.0316270)
Supplement: S9 Table — (DOCX) [file pone.0316270.s014.docx]

**Table S9.** Details of prophage regions of *P. pentosaceus* MBBL6.

| **Prophage region*s* 01: INTAKE** | | | | |
| --- | --- | --- | --- | --- |
| Locus tag | Start | End | Strand | Description |
| V4W90_RS00255 | 53084 | 54157 | - | integrase |
| V4W90_RS00260 | 54526 | 55335 | - | Phage-like_protein |
| V4W90_RS00285 | 57098 | 57508 | - | prophage repressor-like protein |
| V4W90_RS00290 | 57512 | 57859 | - | phage repressor protein |
| V4W90_RS00300 | 58342 | 58542 | + | hypothetical protein |
| V4W90_RS00305 | 58575 | 59378 | + | hypothetical protein |
| V4W90_RS00310 | 59424 | 59624 | + | hypothetical protein |
| V4W90_RS00320 | 60108 | 60854 | + | putative regulatory protein |
| V4W90_RS00325 | 60867 | 61061 | + | Phage-like protein |
| V4W90_RS00345 | 61792 | 62481 | - | domain protein |
| V4W90_RS00350 | 62705 | 63166 | + | hypothetical protein |
| V4W90_RS00355 | 63163 | 63819 | + | ERF-like recombinase |
| V4W90_RS00360 | 63822 | 64220 | + | putative single strand binding protein |
| V4W90_RS00365 | 64231 | 64929 | + | hypothetical protein |
| V4W90_RS00375 | 65094 | 65837 | + | hypothetical protein |
| V4W90_RS00380 | 65818 | 66636 | + | zinc finger protein |
| V4W90_RS00385 | 66753 | 67163 | + | putative Holliday junction resolvase |
| V4W90_RS00405 | 67930 | 68223 | + | hypothetical protein |
| V4W90_RS00410 | 68224 | 68766 | + | hypothetical protein |
| V4W90_RS00430 | 69651 | 69869 | + | Phage-like_protein |
| V4W90_RS00435 | 70145 | 70588 | + | hypothetical protein |
| V4W90_RS00440 | 70830 | 71285 | + | Phage-like_protein |
| V4W90_RS00450 | 71762 | 72226 | + | phage terminase small subunit |
| V4W90_RS00455 | 72226 | 74100 | + | terminase large subunit |
| V4W90_RS00460 | 74301 | 75413 | + | Portal_protein |
| V4W90_RS00465 | 75394 | 75987 | + | protease-scaffold-major head protein |
| V4W90_RS00470 | 75989 | 77329 | + | phage capsid protein |
| V4W90_RS00475 | 77362 | 77685 | + | Phage-like_protein |
| V4W90_RS00480 | 77666 | 78010 | + | putative head-tail joining protein |
| V4W90_RS00485 | 78003 | 78425 | + | putative tail component protein |
| V4W90_RS00490 | 78428 | 78814 | + | putative tail component |
| V4W90_RS00495 | 78814 | 79446 | + | phage tail protein |
| V4W90_RS00505 | 80180 | 84832 | + | putative tail component protein |
| V4W90_RS00510 | 84848 | 85681 | + | minor tail protein |
| V4W90_RS00515 | 85611 | 86834 | + | putative tail endopeptidase |
| V4W90_RS00525 | 87137 | 87424 | + | Hypothetical_protein |
| V4W90_RS00530 | 87424 | 89268 | + | minor capsid protein |
| V4W90_RS00550 | 90510 | 90803 | + | hypothetical protein |
| V4W90_RS00555 | 90803 | 91045 | + | holin |
| V4W90_RS00560 | 91029 | 92153 | + | phage-like_protein |
| **Prophage region*s* 02: INTAKE** | | | | |
| Locus tag | Start | End | Strand | Description |
| V4W90_RS04995 | 244321 | 245337 | - | putative DNA polymerase III |
| V4W90_RS05005 | 245688 | 246317 | - | putative thymidylate kinase |
| V4W90_RS05020 | 247438 | 249183 | - | putative DNA polymerase III |
| V4W90_RS05030 | 249395 | 249880 | - | nucleoside deaminase |
| V4W90_RS05040 | 250690 | 250920 | + | glutaredoxin |
| V4W90_RS05045 | 251022 | 253187 | + | putative ribonucleotide reductase |
| V4W90_RS05050 | 253207 | 254160 | + | putative ribonucleotide reductase |
| V4W90_RS05055 | 254411 | 255397 | - | hypothetical protein |
| V4W90_RS05060 | 255402 | 255833 | - | hypothetical protein |
| V4W90_RS05065 | 256121 | 257245 | - | lysin |
| V4W90_RS05070 | 257229 | 257471 | - | holin |
| V4W90_RS05075 | 257471 | 257758 | - | hypothetical protein |
| V4W90_RS05080 | 257808 | 257954 | - | hypothetical protein |
| V4W90_RS05095 | 259051 | 260886 | - | minor capsid protein |
| V4W90_RS05110 | 261467 | 262603 | - | hypothetical protein |
| V4W90_RS05115 | 262612 | 263457 | - | holin-like putative tail component protein |
| V4W90_RS05120 | 263462 | 268729 | - | minor tail protein |
| V4W90_RS05125 | 268733 | 269365 | - | hypothetical protein |
| V4W90_RS05130 | 269372 | 269809 | - | minor capsid protein |
| V4W90_RS05135 | 270051 | 270491 | - | minor capsid protein |
| V4W90_RS05140 | 270495 | 270890 | - | minor capsid protein |
| V4W90_RS05145 | 270877 | 271227 | - | minor capsid protein |
| V4W90_RS05150 | 271227 | 271574 | - | minor capsid protein |
| V4W90_RS05155 | 271571 | 271987 | - | hypothetical protein |
| V4W90_RS05160 | 272056 | 272967 | - | main capsid protein |
| V4W90_RS05165 | 272980 | 273534 | - | capsid scaffolding protein |
| V4W90_RS05170 | 273634 | 274767 | - | minor capsid protein |
| V4W90_RS05175 | 274764 | 276311 | - | minor capsid protein |
| V4W90_RS05180 | 276314 | 277678 | - | putative terminase large subuni |
| V4W90_RS05185 | 277684 | 278253 | - | terminase small subunit |
| V4W90_RS05200 | 279246 | 279662 | - | ArpU-like protein |
| V4W90_RS05220 | 280588 | 280878 | - | Phage-like_protein |
| V4W90_RS05225 | 280878 | 281207 | - | hypothetical protein |
| V4W90_RS05230 | 281207 | 281569 | - | hypothetical protein |
| V4W90_RS05240 | 282063 | 282587 | - | single-stranded DNA binding protein |
| V4W90_RS05250 | 282834 | 283535 | - | hypothetical protein |
| V4W90_RS05255 | 283539 | 284414 | - | putative replication protein |
| V4W90_RS05260 | 284425 | 285270 | - | hypothetical protein |
| V4W90_RS05265 | 285230 | 285994 | - | Phage-like_protein |
| V4W90_RS05295 | 287545 | 287766 | - | cro-like repressor DNA-binding protein |
| V4W90_RS05300 | 287907 | 288227 | + | putative repressor protein;phage |
| V4W90_RS05305 | 288234 | 288626 | + | hypothetical protein |
| V4W90_RS05310 | 288704 | 289081 | + | putative membrane protein |
| V4W90_RS05315 | 289097 | 290089 | + | putative superinfection immunity protein |
| V4W90_RS05325 | 290514 | 291689 | + | putative phage integrase |
| V4W90_RS05350 | 294283 | 294828 | - | Phage-like_protein |
| **Prophage region*s* 03: INCOMPLETE** | | | | |
| Locus tag | Start | End | Strand | Description |
| V4W90_RS08590 | 10811 | 11734 | - | putative abortive infection bacteriophage resistance protein ORF 37 |
| V4W90_RS08595 | 12783 | 12995 | - | tail lysin |
| V4W90_RS08610 | 14213 | 14638 | - | prophage protein |
| V4W90_RS08620 | 15389 | 15604 | - | Phage-like_protein |
| V4W90_RS08625 | 15945 | 17510 | - | DNA primase |
| V4W90_RS08630 | 17507 | 18286 | - | DNA primase |
| V4W90_RS08645 | 19082 | 19786 | - | putative antirepressor |
| V4W90_RS08655 | 20352 | 20690 | + | CI phage repressor protein |
| V4W90_RS08660 | 20695 | 21105 | + | toxin-antitoxin system |
| V4W90_RS08680 | 23133 | 24284 | + | integrase |
| **Prophage region*s* 04: QUESTIONABLE** | | | | |
| Locus tag | Start | End | Strand | Description |
| V4W90_RS08965 | 5351 | 5938 | - | integrase |
| V4W90_RS08980 | 6770 | 7108 | - | XRE family transcriptional regulator |
| V4W90_RS08990 | 8909 | 9576 | - | integrase core domain protein |
| V4W90_RS09010 | 13609 | 14589 | - | Phage-like protein |
| V4W90_RS09015 | 14570 | 15607 | - | Phage-like protein |
| V4W90_RS09020 | 15964 | 16125 | - | transposase |
| V4W90_RS09025 | 16613 | 17542 | + | transposase |
| V4W90_RS09030 | 17692 | 18706 | + | transposase |
| V4W90_RS09040 | 19406 | 20530 | - | transposase |
