# Supplementary material for: Genomic insights into the probiotic potential and genes linked to gallic acid metabolism in Pediococcus pentosaceus MBBL6 isolated from healthy cow milk
Source: PLoS One. 2024 Dec 26;19(12):e0316270. doi: 10.1371/journal.pone.0316270 (PMC11671016; doi:10.1371/journal.pone.0316270)
Supplement: S10 Table — (DOCX) [file pone.0316270.s015.docx]

**Table S10.** Prediction of CRISPER/Cas in *P. pentosaceus* MBBL6.

| Element | Region | Start | End | Strand |
| --- | --- | --- | --- | --- |
| CRISPER 1 | NZ_JAZIFR010000001_1 | 90,225 bp | 90,304 bp | - |
| CRISPER 2 | NZ_JAZIFR010000001_2 | 369,919 bp | 370,024 bp | + |
| CRISPER 3 | NZ_JAZIFR010000002_1 | 113,521 bp | 113,633 bp | - |
