# Supplementary material for: Genomic insights into the probiotic potential and genes linked to gallic acid metabolism in Pediococcus pentosaceus MBBL6 isolated from healthy cow milk
Source: PLoS One. 2024 Dec 26;19(12):e0316270. doi: 10.1371/journal.pone.0316270 (PMC11671016; doi:10.1371/journal.pone.0316270)
Supplement: Attachment — Submitted filename: Response to Reviewers.pdf [file pone.0316270.s017.pdf]

Point-by-point responses to the reviewers' comments

Reviewer # 1

Reviewer comment: I commend the comprehensive investigation of *P.pentosaceus* MBBL6 and its potential as a probiotic. Your findings are outstanding. The paper is well-structured, with a logical flow that guides the reader through the research objectives, methodology, and findings.

Our Response: We sincerely thank you for the encouraging comments and valuable remarks on the manuscript. Your encouraging words are greatly appreciated and motivate us to continue exploring innovative solutions in probiotic research.

Reviewer ## 2

Reviewer comment: The manuscript demonstrates a whole genome analysis of *P. pentosaceus* MBBL6 (isolated from healthy cow's milk) to study its probiotic potential. The study presented is a comprehensive one that shows the involved metabolic pathways, carbohydrate-active enzymes, bacteriocin gene clusters, and secondary metabolite biosynthetic gene clusters. The manuscript could do a better job of describing the strain-specific genes (and associated pathways) to emphasize the significance of MBBL6, based on comparison with closely related genomes. Next, the manuscript could also discuss how the results compare with other strains (for example, Kompramool S., et al, 2024) that have also demonstrated antimicrobial activity. I recommend that the manuscript be published once these abovementioned have been addressed. Overall, the distinct or unique molecular functions of the strain based on a comparative analysis could be discussed further.

Our Response: Thank you for your encouraging comments on our work and for your valuable suggestions to improve the manuscript. *P. pentosaceus* MBBL6 possesses several specific genes and pathways, including bacteriocin production (e.g., bovicin_255 and penocin A), primary metabolic regions for gallic acid and arginine to hydrogen carbonate metabolism, secondary metabolites (e.g., RiPP, T3PKS), important carbohydrate-active enzymes, and riboflavin biosynthesis. In response to your suggestion, we have also included a comparative discussion with other strains, including the findings of Kompramool et al. (2024), to contextualize our results

within the broader landscape of *P. pentosaceus* strains demonstrating antimicrobial activity. Furthermore, for the first time, we report that bovicin strongly interacts with the Rho factor, a protein involved in terminating transcription and essential for playing a critical role in regulating gene expression and ensuring proper RNA synthesis. The binding of bovicin to these proteins suggests a potential mechanism by which MBBL6 exerts its inhibitory effects, potentially disrupting the transcription termination process and thereby hindering the survival and proliferation of mastitis pathogens such as *S. aureus* D4C4 and *E. coli* G1C5. This addition will further highlight the unique molecular functions and probiotic potential of MBBL6. Thank you again for your valuable recommendations, which will undoubtedly strengthen the manuscript. Please refer to the revised manuscript.

Reviewer ### 3

Reviewer comment: What are the main reasons for selecting *Pediococcus pentosaceus* MBBL6 isolated from healthy cow milk at BSMRAU dairy farm, Gazipur, Bangladesh, as the study subject?

Our Response: Thank you for your question. *Pediococcus pentosaceus* is recognized for producing antimicrobial compounds like pediocin, a bacteriocin with broad-spectrum activity against bacterial pathogens, including *Escherichia coli*, *Staphylococcus aureus*, *Bacillus subtilis*, *Klebsiella pneumoniae*, *Pseudomonas aeruginosa*, *Enterococcus faecalis*, *Acinetobacter baumannii*, as well as *Listeria* and *Clostridium* species (Diep et al. 2006; Kompramool et al. 2024; Kuniyoshi et al. 2022; Thao et al. 2021; Zaghloul and Halfawy 2024). Therefore, we aimed to isolate and sequence *P. pentosaceus* MBBL6 assuming its probiotic potential, antimicrobial activity, and relevance to dairy health. Isolated from healthy cow milk at BSMRAU dairy farm, it shows promise for mastitis control as evidenced by both *in-vitro* and *in-vivo* experiments.

Reviewer comment: What are the main objectives of the comprehensive genomic analysis of *P. pentosaceus* MBBL6 in this study?

Our Response: Thank you for your question. This study aimed to sequence and analyze the genome of *P. pentosaceus* MBBL6, isolated from healthy cow's milk, to elucidate its genomic characteristics, safety profile, antimicrobial efficacy, and probiotic potential. The ultimate goal of this study was to isolate a potential probiotic for the development of biorational therapeutics

targeting bovine mastitis pathogens. You may kindly go through **Lines 98 – 103** in the revised manuscript.

Reviewer comment: What methods were used in this study to evaluate the probiotic and antimicrobial potential of *P. pentosaceus* MBBL6, both *in-vitro* and *in-vivo*?

Our Response: We appreciate the reviewer query. The MBBL6 strain was evaluated for its probiotic potential against two major pathogens of bovine mastitis such as *S. aureus* and *E. coli*, using both *in-vitro* (agar well diffusion technique) and *in-vivo* (mouse mastitis model) methods. In brief, agar well diffusion method was performed using concentrated cell-free supernatant (CFS) of MBBL6. Simultaneously, inoculums (10^6 CFU/mL) of *S. aureus* and *E. coli* were spread on nutrient agar plates (Oxoid, UK). Several wells (6 mm in diameter) were made in each nutrient agar plate and filled with 100 µL of CFS from MBBL6. Following a 24 h incubation period at 37°C, the antibacterial activity was assessed by measuring the diameter of the inhibition zones. To investigate the antimicrobial effectiveness of MBBL6 *in-vivo*, we conducted a mouse mastitis model experiment using 20 germ-free Swiss albino timed pregnant mice (Day 18 of breeding), divided into two groups: Group I (n=10) and Group II (n=10), following our previously published protocol (Hoque et al. 2024; Hoque et al. 2022). You may kindly refer to **Lines 205 – 222** in the revised manuscript.

Reviewer comment: What are the main findings related to genes involved in carbohydrate metabolism and vitamin B complex biosynthesis in the genome of *P. pentosaceus* MBBL6?

Our Response: Thank you for your question. By analyzing the KEGG metabolic pathways we found that MBBL6 genome contained complete set of genes for glucose (e.g., *glk*, *gapA*, *gpmA*, *eno* etc.), galactose (e.g., *galM*, *galK*, *galT* etc.) pentose phosphate (e.g., *pfkA*, *rbsK*, *prsA*, *rpiA* etc.) metabolism. Additionally, *lacZ* (beta-galactosidase) found in MBBL6 genome which involved in converting lactose to glucose and galactose. Further analysis revealed that the MBBL6 strain harbors several genes associated with vitamin B-complex biosynthesis. Notably, genes such as *ribA/B/D/E/F/H*, *thiM*, *ybjI*, *nfrA1*, *ubiX* etc. identified in MBBL6, were found to be involved in the riboflavin biosynthesis pathway (M00125). Additionally, these genes have the potential to reduce gut inflammation through immunomodulation and contribute to maintaining gut microbiota homeostasis (Zhang et al. 2024). You may kindly go through **Lines 321 – 330, 349 – 352** and **500 – 501** in the revised manuscript.

Reviewer comment: How do the results of *in-vivo* tests in a mouse mastitis model support the therapeutic potential of *P. pentosaceus* MBBL6 in overcoming mastitis pathogens?

Our Response: Thank you for your query. Although there are differences between mice and larger mammals, the basic immune responses and mechanisms of infection and inflammation in the mammary gland are similar. The *in-vivo* tests through a mouse mastitis model demonstrated that *P. pentosaceus* MBBL6 effectively suppressed mastitis pathogens such as *S. aureus* D4C4 and *E. coli* G1C5, supporting its therapeutic potential. The strain exhibited significant antimicrobial activity, reducing infection severity and alleviating inflammation in the model. These results underscore the promising role of MBBL6 as a potential probiotic treatment for mastitis, further validating its efficacy observed in *in-vitro* studies. You may kindly go through **Lines 572 – 578** in the updated manuscript.

Reviewer comment: What are the main conclusions that can be drawn from the genomic analysis of *P. pentosaceus* MBBL6 related to its applications in therapy, bioremediation, and biopreservation?

Our Response: We do appreciate the reviewer for such a nice query. The genomic analysis of *P. pentosaceus* MBBL6 highlights its genomic features, safety profile, antimicrobial efficacy, and probiotic potential. Its antimicrobial properties, including bacteriocin production, suggest therapeutic use for mastitis and other infections. Gallic acid and its derivatives are recognized for their numerous health benefits, including promoting gut microbiome homeostasis, antioxidant, antimicrobial, anticancer, anti-inflammatory, gastroprotective, cardioprotective, neuroprotective effects, and prevention of metabolic diseases. Notably, *P. pentosaceus* MBBL6, identified as a probiotic strain, exhibits the ability to metabolize gallic acid, highlighting its potential for applications in biopreservation and bioremediation.

Reviewer comment: Use a minimum of the last 5 years of literature, especially research articles.

Our Response: Thank you for your valuable suggestion. We have updated the manuscript by incorporating relevant research articles from the last five years to ensure the most current literature is referenced. This has enabled us to enhance the discussion and offer a more current context for the findings presented in our study. Please refer to the updated manuscript.
